# Intensive Knee Chest Upper Cervical Chiropractic Care in a Traveling Patient With Migraine: A Case Report

**DOI:** 10.7759/cureus.117366

**Published:** 2026-10-02

**Authors:** Jessey Wong, Shaan Rai

**Affiliations:** 1 Chiropractic, Vitality Chiropractic Centres, Singapore, SGP

**Keywords:** case report, cervicogenic headache, chiropractic singapore, chiropractor singapore, cluster headache, knee chest upper cervical technique, migraine, status migrainosus, upper cervical chiropractic, vertebral subluxation

## Abstract

Migraine and cluster headache are disabling primary headache disorders that may respond to upper cervical intervention via shared trigeminocervical mechanisms, though clinical evidence for this approach in migraine specifically remains limited and mixed. We report a 46-year-old female program coordinator, resident in Sri Lanka, with a 10-year history of migraine who developed a new, daily headache pattern that she described as "cluster headache", a label of uncertain clinical provenance reported here as an unconfirmed differential. She was also hospitalized for two nights with a two-week headache exacerbation, vomiting, and blurred vision, discharged with a diagnosis of status migrainosus after morphine and an occipital nerve block. About one week after discharge, she developed transient neurological symptoms, including confusion and memory difficulty, without contemporaneous imaging workup. Examination and imaging identified postural asymmetry and a C1 subluxation complex. Because knee chest upper cervical (KCUC) chiropractic is unavailable in her home country, she traveled to Singapore for an intensive, twice-daily care model over six days and then three visits at the four-month follow-up. Baseline Bournemouth Questionnaire score was 37/70. By the end of the intensive week, examination findings had normalized, with resolution of photophobia and phonophobia and regained occipital sensation. At the four-month follow-up, she reported approximately 95% headache improvement and resolution of cognitive and visual symptoms, with no adverse events. This case illustrates a temporal association between intensive KCUC care and improvement in a patient with migraine, status migrainosus, and a self-described cluster headache-like pattern and highlights considerations for chiropractic care delivered to patients traveling internationally for care unavailable at home.

## Introduction

Cluster headache is an excruciating, strictly unilateral primary headache disorder accompanied by ipsilateral cranial autonomic features, while migraine is a common, chronic disorder characterized by recurrent, disabling attacks; both are classified according to criteria set out in the International Classification of Headache Disorders, 3rd edition (ICHD-3) [1]. Cluster headache in particular carries a substantial burden on quality of life and can be difficult to manage with pharmacological therapy alone [2].

It is important to note at the outset that migraine and cluster headache are distinct diagnostic entities under ICHD-3 criteria, with different attack durations, periodicity, and associated features; where this report later uses a patient's own description of her symptoms as "cluster headache", this reflects her own terminology only and does not imply diagnostic equivalence with, or endorsement of, a formal cluster headache diagnosis.

A body of basic-science literature describes convergence of nociceptive afferents from the trigeminal system and the upper cervical spine onto second-order neurons of the trigeminocervical complex, providing a physiological rationale for why irritation or dysfunction in the upper cervical spine may contribute to, or modulate, headache presentations that are classified as primary in origin [3]. The clinical evidence supporting manual or chiropractic intervention for migraine specifically remains limited and mixed, and this rationale should be read as a physiological plausibility argument rather than an evidence-based indication. This convergence has motivated interest in manual and chiropractic interventions directed at the cervical spine for headache disorders, although the clinical evidence base for this approach remains an area of active study and is discussed further below.

We report a case in which a patient with a long-standing history of migraine was diagnosed with status migrainosus during a hospital admission and separately described her headache pattern using the term "cluster headache", a label whose clinical provenance (a treating clinician versus the patient's own research) could not be confirmed and is therefore reported here as an unconfirmed differential rather than an established diagnosis. Her course was further complicated by a serious hospitalization and a constellation of transient neurological symptoms. She subsequently traveled from her home country, Sri Lanka, to Singapore for knee chest upper cervical (KCUC) chiropractic care, a modality not available where she lives. Because of this, care was delivered in a condensed, twice-daily intensive format that differs from the frequency typically described in prior KCUC case reports. This case is reported in accordance with the CARE (CAse REport) guidelines to support transparent and complete reporting [4].

## Case presentation

A 46-year-old female program coordinator, resident in Sri Lanka, presented to a Singapore-based upper cervical chiropractic clinic with a 10-year history of migraine and a three-month history of a new, daily headache pattern. She traveled specifically to Singapore for this care, as KCUC chiropractic is not available in her home country. She described sharp pain behind the left eye for the preceding 1.5 months, throbbing pain at the crown of the head, and frontal tension, with marked intolerance of light and sound. She described this new pattern using the term "cluster headache"; whether this label reflected an assessment by a treating clinician or the patient's own research could not be confirmed from the available records, and it is reported here as an unconfirmed differential rather than an established diagnosis (see Diagnosis and Limitations). During the same general period, she was also hospitalized under her treating consultant neurologist with a final diagnosis of status migrainosus (detailed below); this diagnosis, together with her decade-long background of migraine and the unconfirmed cluster headache label, is carried forward as the working differential for this case (see Diagnosis and Discussion).

Hospitalization (day -30 to day -28)

The patient was admitted to a private hospital in Sri Lanka on day -30 under the treating consultant neurologist, with a two-week history of headache that had worsened on the morning of admission, one episode of vomiting, and blurred vision; no other associated neurological symptoms were documented at the time of admission. On examination, she was not pale and afebrile, with a blood pressure of 165/109 mmHg, a pulse rate of 91 bpm (regular), a respiratory rate of 18/min, an oxygen saturation of 99% on room air, a Glasgow Coma Scale (GCS) score of 15/15, bilateral pupils equal and reactive to light, no focal neurological signs, and a soft, non-tender abdomen. She was managed with intravenous paracetamol, intravenous ondansetron, intravenous dexamethasone, subcutaneous morphine (2.5 mg eight-hourly for three doses, increased to 5 mg eight-hourly for two further doses), and reduced doses of her usual topiramate and melatonin. On day -29, the consultant neurologist performed a bilateral greater and lesser occipital nerve block using intramuscular Depo-Medrol (methylprednisolone) 40 mg to each side with 2% lignocaine. An electrocardiogram (ECG) performed on day -28 showed no acute ischemic changes. She was discharged on day -28 in a clinically stable condition with a final diagnosis of status migrainosus, on zolmitriptan 2.5 mg PRN, ondansetron 4 mg PRN, topiramate 50 mg nocte, melatonin 9 mg nocte, and a one-week oral dexamethasone taper (18 mg once daily) with esomeprazole 20 mg twice daily for gastric cover, with a follow-up plan to review with the treating consultant neurologist as needed.

Approximately one week after this discharge (around day -21), she developed a two-week period of confusion and memory difficulty. She also reported transient difficulty distinguishing blue from green (an optician's assessment was negative for an ocular cause), intermittent speech difficulty with tongue fatigue and thirst, and bilateral hand and arm weakness (e.g., difficulty opening a bottle). No neurological symptoms of this kind were documented at the time of the hospitalization itself. Brain MRI performed approximately two years before day 0 had excluded ischemic changes, and head and neck MRI and CT performed approximately one year before day 0 had been unremarkable; both predate this hospitalization and the subsequent neurological symptoms and are reported here only as relevant prior imaging history, not as workup of the episode itself (see Diagnostic imaging and Limitations). These findings are described in this report as part of the patient's medical history; they were managed by her treating medical team independently of the chiropractic care described below, and their resolution is not attributed to the intervention described in this report (see Discussion).

Current medications at presentation were zolmitriptan 2.5 mg and topiramate 50 mg, used for headache. Separately from the hospitalization described above, she reported an ongoing history of sports-medicine physiotherapy massage/exercise and acupuncture, used sporadically over an unspecified period with her most recent sessions in the two weeks before presentation, and Ayurvedic and head-oil massage used approximately monthly over a longer, unspecified duration; the exact frequency and duration of these treatments could not be established, and the Ayurvedic massage was reportedly used for general wellness and possibly sinus symptoms in addition to headache. She reported limited or transient benefit from these approaches. She reported no relevant surgical, family, or substance-use history and described herself as physically active (walking). A timeline of key clinical events is presented in Table 1.

**Table 1:** Clinical timeline. IM: intramuscularly; ECG: electrocardiogram; BQ: Bournemouth Questionnaire; KCUC: knee chest upper cervical

Timepoint	Clinical event
Approximately 10 years before day 0	Onset of episodic migraine headache
Approximately 2 years before day 0	Brain MRI performed: reported as excluding ischemic changes. This predates and does not represent workup for the acute neurological episode described below
Approximately 1 year before day 0	Head and neck MRI and CT performed: reported as clear. As with the earlier brain MRI, this predates the acute episode described below and does not represent contemporaneous investigation of it
Approximately 3 months before day 0	Onset of new daily headache pattern (sharp left retro-orbital pain, crown/vertex throbbing, frontal tension, photophobia, phonophobia), which the patient described using the term "cluster headache"; the clinical provenance of this label (clinician-given versus patient's own research) could not be confirmed (see Diagnosis and Limitations)
Day -30 to day -28	Hospitalized at a private hospital in Sri Lanka under the treating consultant neurologist with a 2-week headache exacerbation, one episode of vomiting, and blurred vision; no other neurological symptoms documented at admission. Treated with IV paracetamol, IV ondansetron, IV dexamethasone, and subcutaneous morphine (2.5 mg 8-hourly ×3 and then 5 mg 8-hourly ×2). Bilateral greater/lesser occipital nerve block (IM Depo-Medrol 40 mg ×2 with 2% lignocaine) performed on day -29. ECG on day -28: no acute ischemic changes. Discharged on day -28, clinically stable, with a final diagnosis of status migrainosus, on a tapering course of oral dexamethasone and esomeprazole plus maintenance zolmitriptan, ondansetron, topiramate, and melatonin
Approximately day -21 to day -7	Two-week period of confusion and memory difficulty, beginning approximately one week after discharge (around day -21); transient difficulty distinguishing blue from green (ophthalmology review negative); intermittent speech/tongue fatigue and thirst; bilateral hand/arm weakness (e.g., difficulty opening bottles). No neuroimaging or other investigation specific to these symptoms is documented; the only available brain and head/neck imaging (approximately 2 and 1 years before day 0, above) predates them and cannot be considered a contemporaneous workup (see Limitations)
At other times before day 0	Reported an ongoing history of sports-medicine physiotherapy massage/exercise and acupuncture, used sporadically with her most recent sessions in the 2 weeks before presentation (exact prior frequency/duration unknown), and Ayurvedic/head-oil massage used approximately monthly over a longer, unspecified duration for general wellness and possibly sinus symptoms as well as headache
Day 0	Initial presentation to Vitality Chiropractic Centres. Baseline history, postural, orthopedic/neurological examination, cervical radiographs, and infrared thermography performed. Baseline BQ score: 37/70
Days 0-5	Intensive KCUC care, twice daily, guided by twice-daily thermographic reassessment (10 visits over 6 days)
10th visit (day 5, end of intensive week)	Re-examination showed balanced shoulder height and head tilt, even leg length, normalized weight distribution, resolution of left-eye red desaturation, and normal smooth pursuit. The patient reported regained sensation in the occipital region (previously lost following nerve block), reduced headache, improved tolerance of phone use, and resolution of photophobia/phonophobia
Approximately 4 months after day 0	The patient returned to Singapore for a follow-up course of 3 visits over 5 days. Reported approximately 95% reduction in headache; improvement in brain fog/concentration difficulty (noted approximately 2 weeks after the completion of the intensive program) and improved color discrimination
Discharge	The patient transitioned to a full-spine chiropractor in Sri Lanka for maintenance care

Physical examination

Postural observation revealed a high right shoulder and a right head tilt. Cervical range of motion showed pain on bilateral lateral flexion, bilateral rotation, and extension. Cervical distraction and compression tests were negative. Weight-scale analysis showed 27.65 kg borne on the left and 30.75 kg on the right, a 3.1 kg right-sided lean. Weight distribution was assessed using a bilateral weight-scale analysis, in which the patient stands with one foot on each of two adjacent scales, allowing direct comparison of weight borne on each side as an indicator of postural/weight-bearing asymmetry. Upper limb muscle testing showed weakness of the left arm on right cervical flexion. A right short leg was noted on prone leg length check, with a positive finding on right head rotation (a method similar in principle to the Derifield-Thompson leg check). The red desaturation test was positive in the left eye, and the right eye showed saccadic movement on horizontal and vertical smooth pursuit testing. The red desaturation test compares the perceived color saturation of a red target presented separately to each eye, with reduced saturation in one eye considered a positive finding suggestive of afferent visual pathway asymmetry. Smooth pursuit was assessed by having the patient visually track a slowly moving target, with breakdown into saccadic movement considered a positive finding suggestive of oculomotor or vestibular dysfunction. Both tests were performed by a single examiner using routine clinical observation rather than a standardized instrument or validated scoring protocol. Given these findings in the context of her unexplained post-discharge neurological symptoms, the patient was verbally advised to follow up with her neurologist; as she already had an established treating neurologist, no formal referral letter was issued by the chiropractic team.

Diagnostic imaging

Cervical radiographs obtained on day 0 comprised five standard views: lateral cervical (Figure 1), base posterior (Figure 2), and anteroposterior open-mouth (APOM) views in neutral (Figure 3), left lateral flexion (Figure 4), and right lateral flexion (Figure 5). These demonstrated loss of the normal cervical lordosis with straightening of the cervical spine, mild osteophytosis of the vertebral bodies, and minimal narrowing of the C5/6 disc space, without loss of vertebral height. The atlanto-axial articulation was unremarkable, and the lateral atlanto-dental intervals were symmetrical bilaterally. The patient's history also included a brain MRI (approximately two years before day 0), reported as excluding ischemic changes, and a head and neck MRI/CT (approximately one year before day 0), reported as clear. Both of these studies predate, by one to two years, the hospitalization and subsequent neurological symptoms (confusion, memory difficulty, and other symptoms) described in the Case Presentation, and neither was obtained to investigate that episode; no neuroimaging specific to that episode is documented in the available records. Infrared thermography was performed at baseline and used, alongside postural and orthopedic findings, as an adjunct clinical tool to guide the timing of adjustments during the intensive care period. Thermography is also used at the treating clinic as a clinical outcome measure, and serial images are presented descriptively in this report for that purpose; however, the research evidence supporting thermography as a validated, reliable outcome measure for headache disorders remains thin, including reported limitations in the inter-examiner reliability of thermographic pattern interpretation in chiropractic practice [5], and no quantitative thermographic findings are reported here.

**Figure 1:**
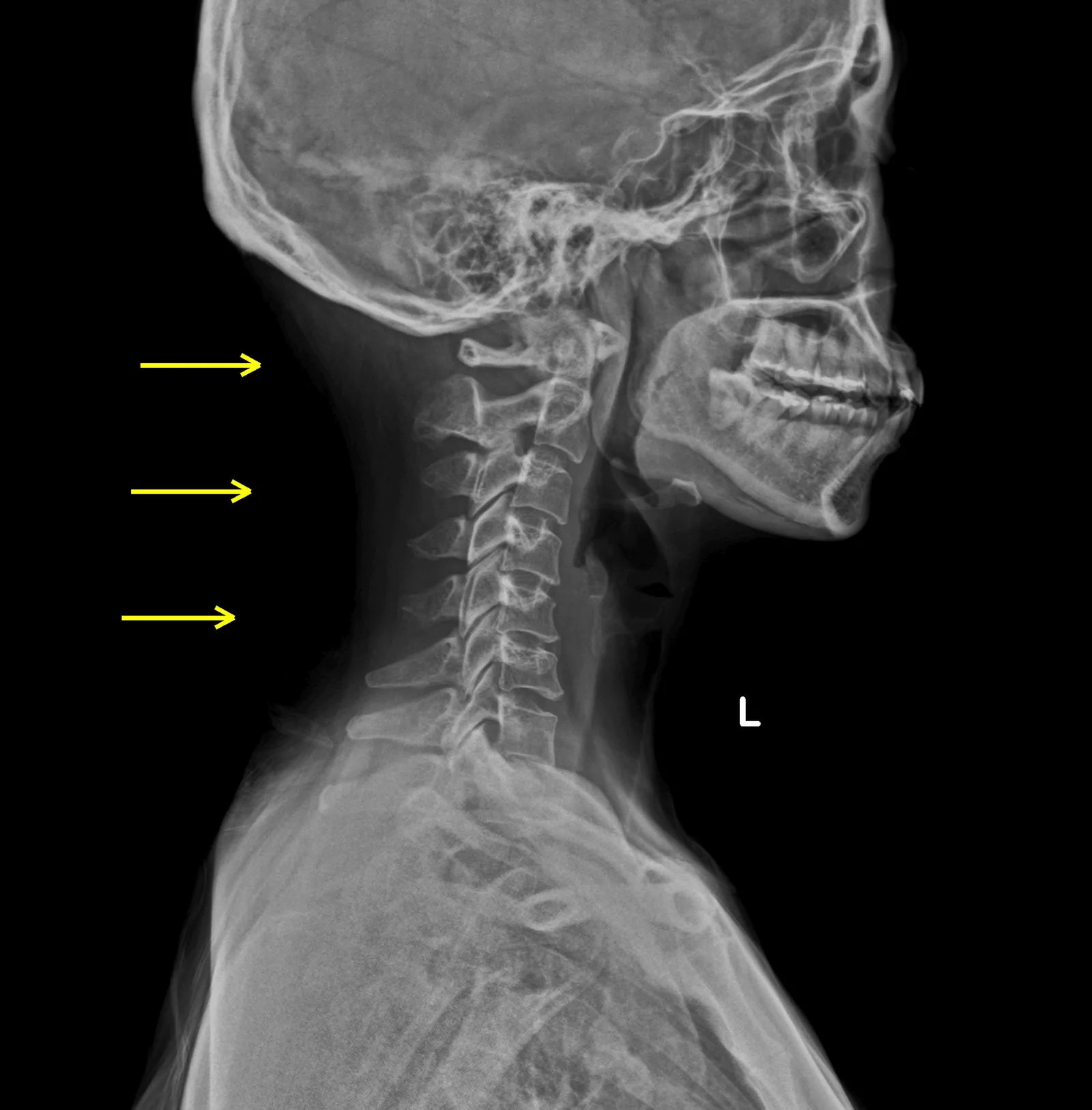
Lateral cervical radiograph depicting loss of normal cervical lordosis and straightening of the cervical spine. Arrows indicate the cervical spine, highlighting the absence of normal lordotic curve.

**Figure 2:**
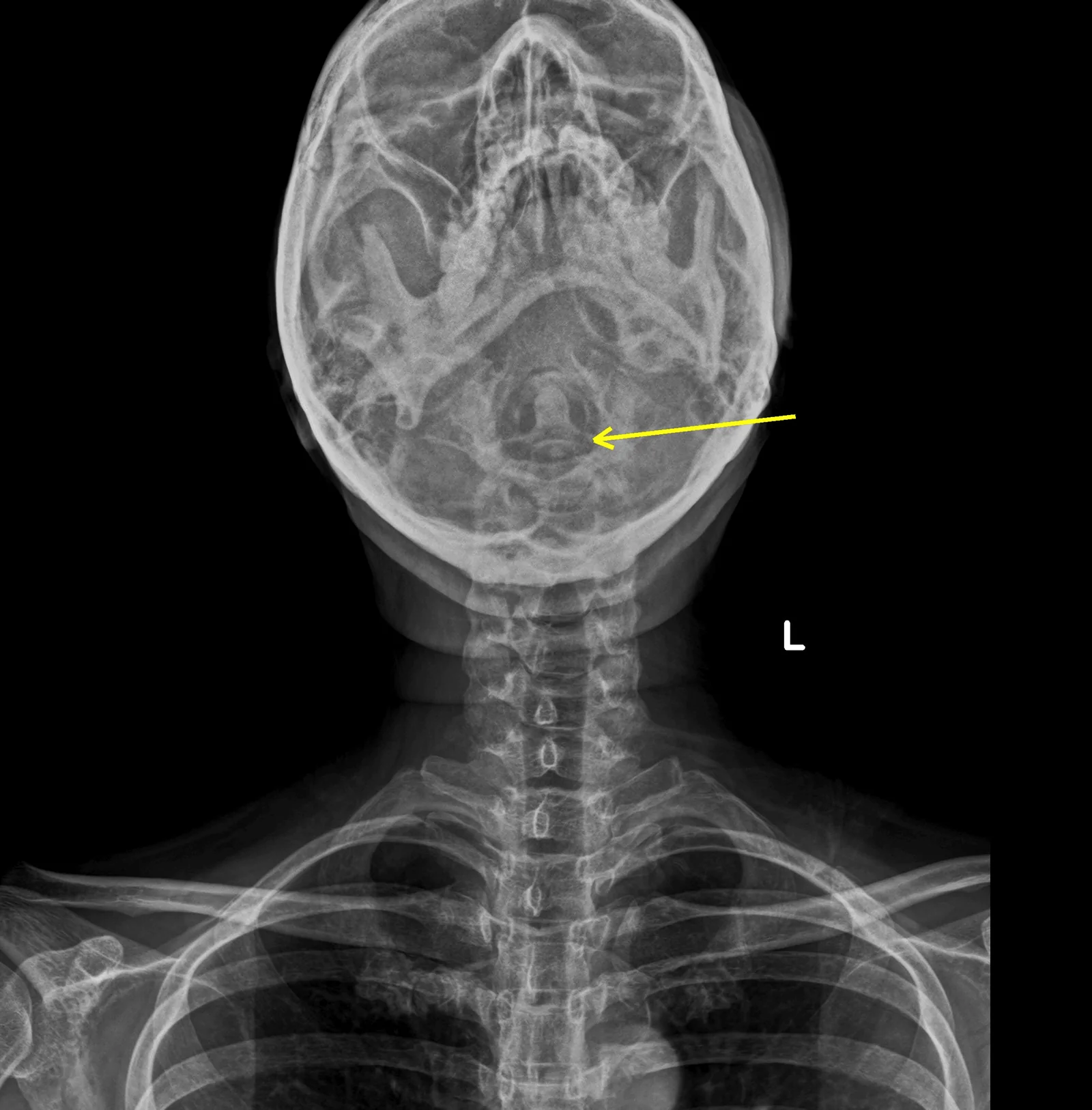
Base posterior (vertex) radiograph. Arrow indicates the upper cervical joint region.

**Figure 3:**
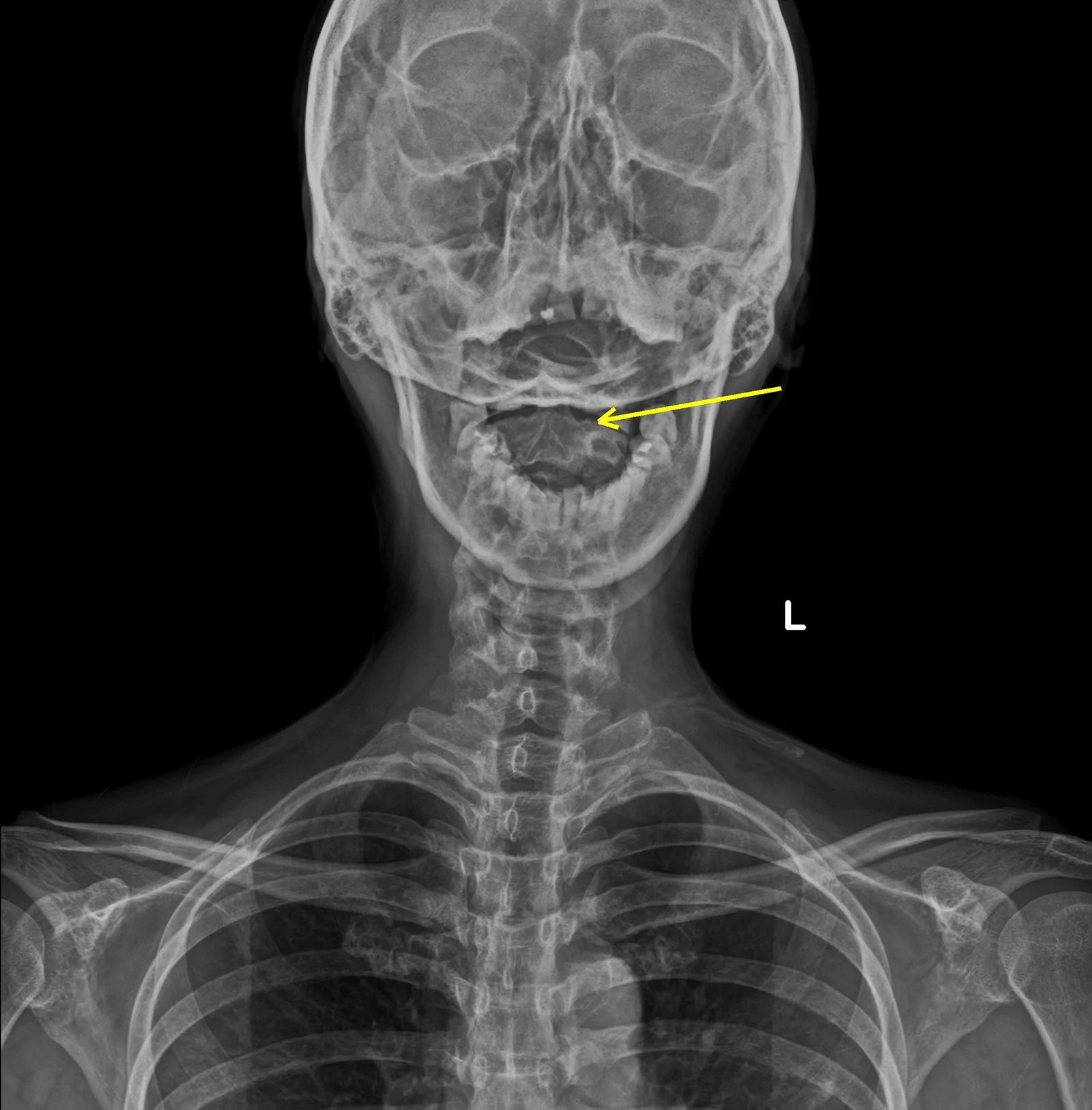
Anteroposterior open-mouth radiograph in neutral position. Arrow indicates the upper cervical region.

**Figure 4:**
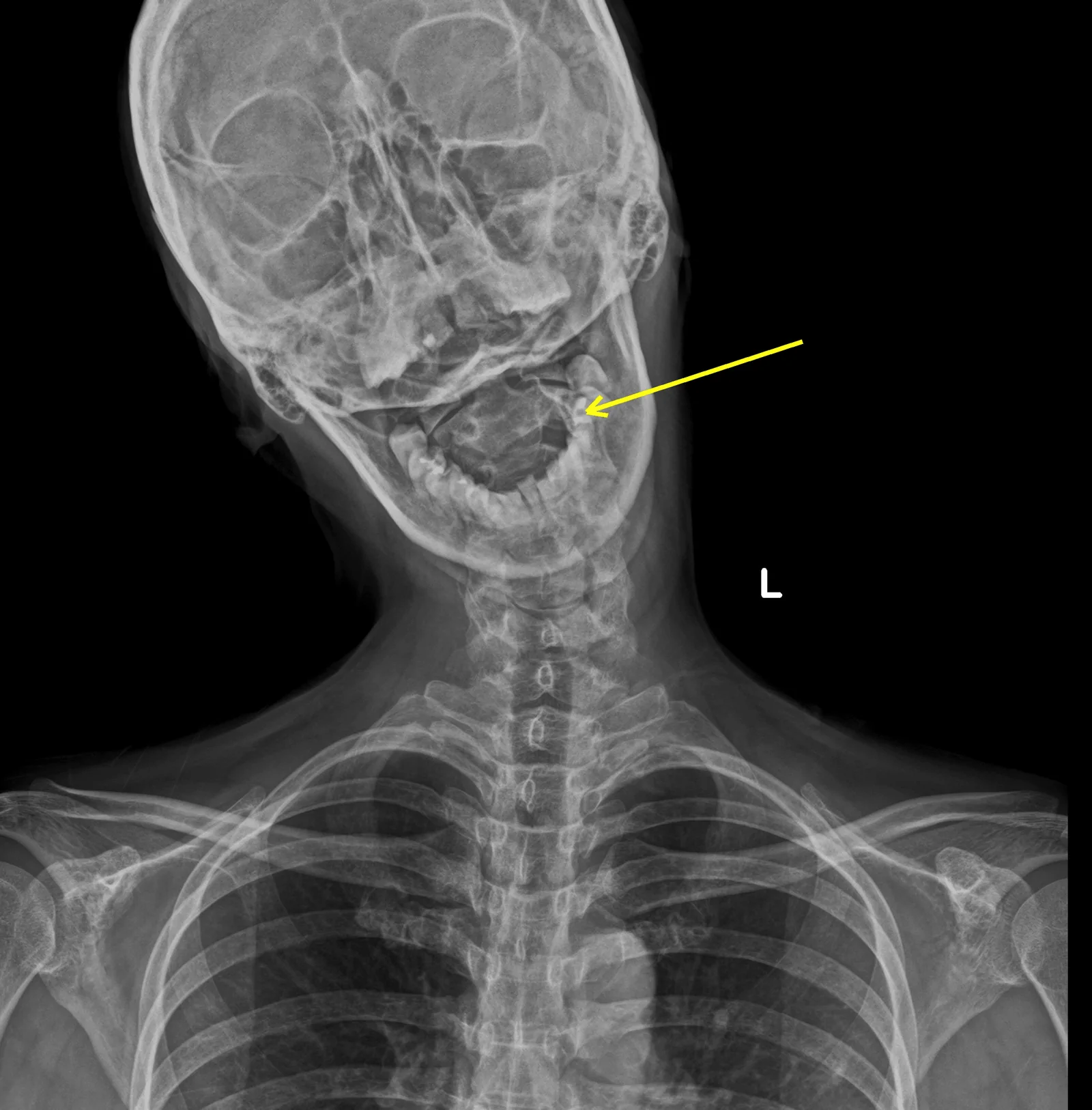
Anteroposterior open-mouth radiograph in right lateral flexion. Arrow indicates the upper cervical region.

**Figure 5:**
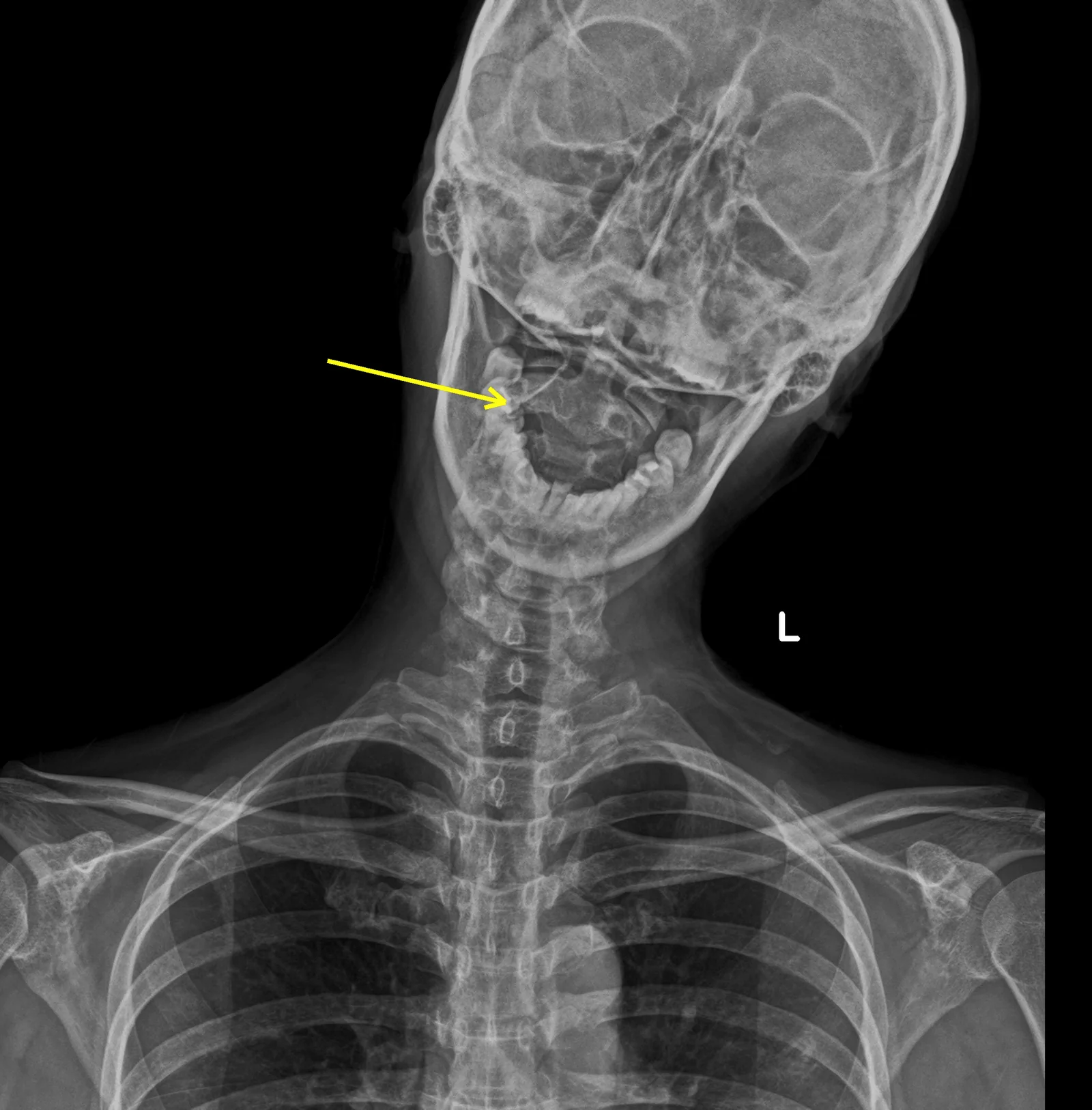
Anteroposterior open-mouth radiograph in left lateral flexion. Arrow indicates the upper cervical region.

Diagnosis

Based on postural, orthopedic, and radiographic findings, the chiropractic diagnosis was a vertebral subluxation complex at C1. The patient's working medical diagnosis was more complex. During a two-night hospital admission (day -30 to day -28) for a two-week headache exacerbation with vomiting and blurred vision, her treating consultant neurologist's discharge diagnosis was status migrainosus [6]; this diagnosis is documented in the hospital discharge summary and is treated in this report as confirmed. Separately, the patient described her three-month pattern of daily, left-sided headache using the term "cluster headache"; the clinical provenance of this label, whether it originated from a treating clinician or from the patient's own research, could not be established from the available records. Formal ICHD-3 diagnostic criteria for cluster headache, attack duration of 15-180 minutes, characteristic attack frequency and periodicity, and ipsilateral cranial autonomic features, were not documented as having been assessed or met in this patient, which is a further reason this label is treated as an unconfirmed differential rather than a diagnosis. It is therefore also reported here as an unconfirmed differential rather than a diagnosis rendered by any treating clinician, and it is not treated as equivalent in evidentiary weight to the documented status migrainosus diagnosis. Both possibilities are carried forward, together with the patient's 10-year history of migraine, as the working differential for this case; formal headache-specific classification testing beyond clinical history and examination was not documented as having been performed. None of these diagnoses or labels were rendered by the chiropractic authors.

Treatment

The patient underwent KCUC chiropractic care, a technique in which a high-velocity, low-amplitude thrust is delivered to a specific upper cervical vertebra with the patient positioned prone in a knee-chest posture and the head rotated to one side. Listings were initially determined by postural analysis, leg-length assessment, muscle testing, balance assessment, and infrared thermography and were confirmed radiographically prior to the first adjustment.

Because the patient had traveled from Sri Lanka to Singapore specifically for this course of care, KCUC not being available in her home country, treatment was delivered in an intensive, condensed format rather than the once-daily or several-times-weekly frequency more typically described in KCUC case series. Over the first six days (days 0-5), she received 10 visits at a frequency of approximately twice daily, with thermographic reassessment performed before each visit to determine whether a further adjustment was indicated (Figure 6 shows serial readings from day 0). No changes were made to this care plan during the intensive period. Approximately four months later, the patient returned to Singapore for a second, shorter course of three visits over five days. In total, she received 13 visits.

**Figure 6:**
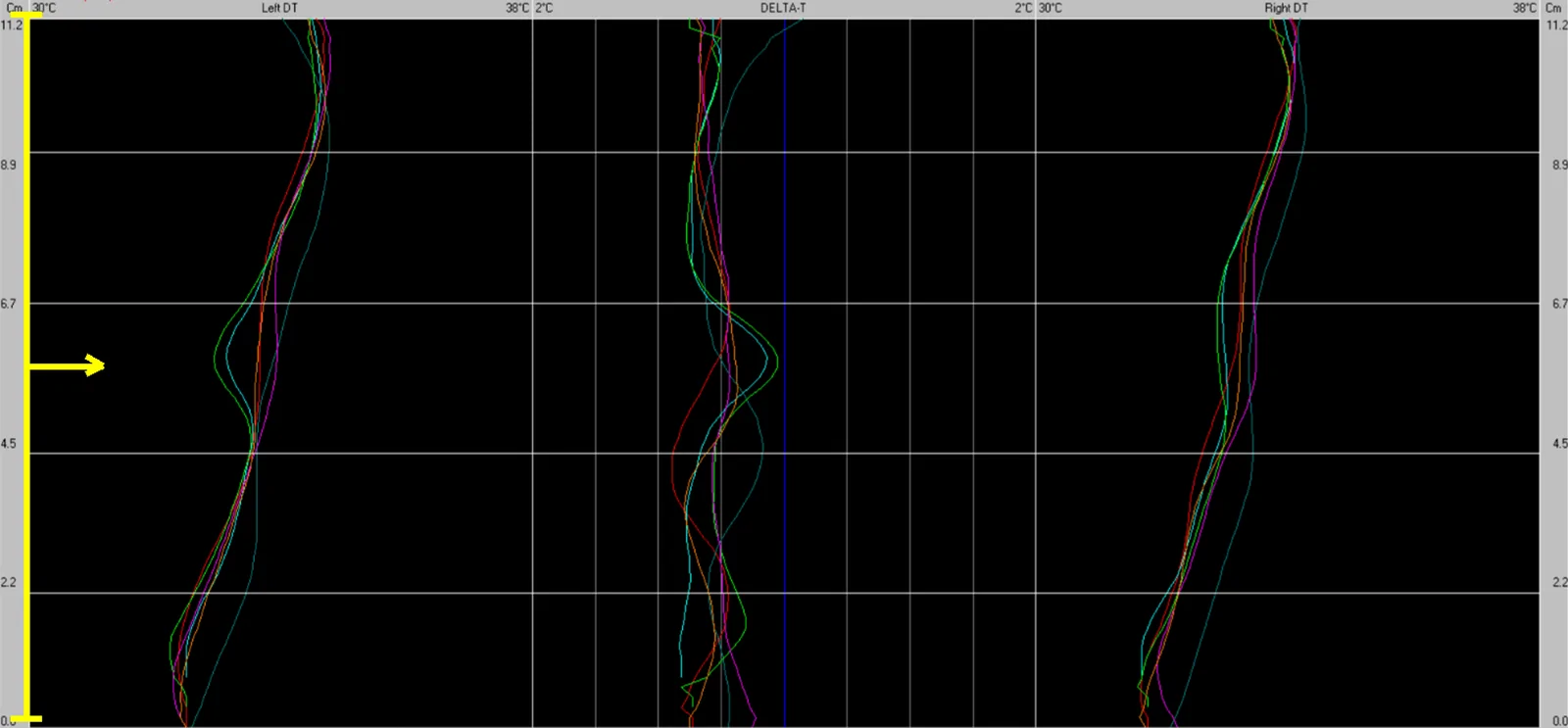
Paraspinal infrared thermography at initial consultation (day 0). Bracket indicates the full scan area assessed for deviation from delta, total width difference in paraspinal temperature, and baseline establishment. Dates and practitioner comment text have been redacted to protect confidential patient information.

During visits when thermographic reassessment did not indicate that a further adjustment was warranted, the patient was instead guided through functional neurological rehabilitation exercises, including vestibulo-ocular reflex exercises (VOR1 and VOR2), figure-8 arm movements, and laser-guided head movement drills, which she performed standing. These exercises constituted a concurrent intervention delivered alongside KCUC adjustments; their independent contribution to the outcomes reported below cannot be isolated from that of the chiropractic care itself (see Discussion).

Outcome and follow-up

At baseline, the patient's Bournemouth Questionnaire (BQ) [7] score was 37/70 (day 0). A discharge or follow-up BQ score was not obtained, as the patient was not formally re-tested using this instrument before returning to Sri Lanka; this is addressed further under Limitations. Objective examination findings at the 10th visit, compared with baseline, are summarized in Table 2. Corresponding thermographic images at baseline and at the end of the intensive care week are shown in Figure 7.

**Table 2:** Baseline versus 10th-visit examination findings. AROM: active range of motion

Finding	Baseline (day 0)	10th visit
Postural observation	High right shoulder; right head tilt	Balanced shoulder height and head tilt
Leg length	Right short leg	Even bilaterally
Weight distribution (scale)	27.65 kg left/30.75 kg right (3.1 kg right lean)	30.25 kg left/30.0 kg right (0.75 kg right lean)
Cervical AROM	Bilateral lateral flexion, rotation, and extension painful	Bilateral lateral flexion painful; rotation and extension improved
Upper limb muscle testing	Left arm weak on right cervical flexion	No abnormality detected
Red desaturation test (left eye)	Positive	Negative
Smooth pursuit (right eye)	Saccadic on horizontal and vertical testing	No abnormality detected
Bournemouth Questionnaire	37/70 (day 0)	Not re-administered (see Limitations)

**Figure 7:**
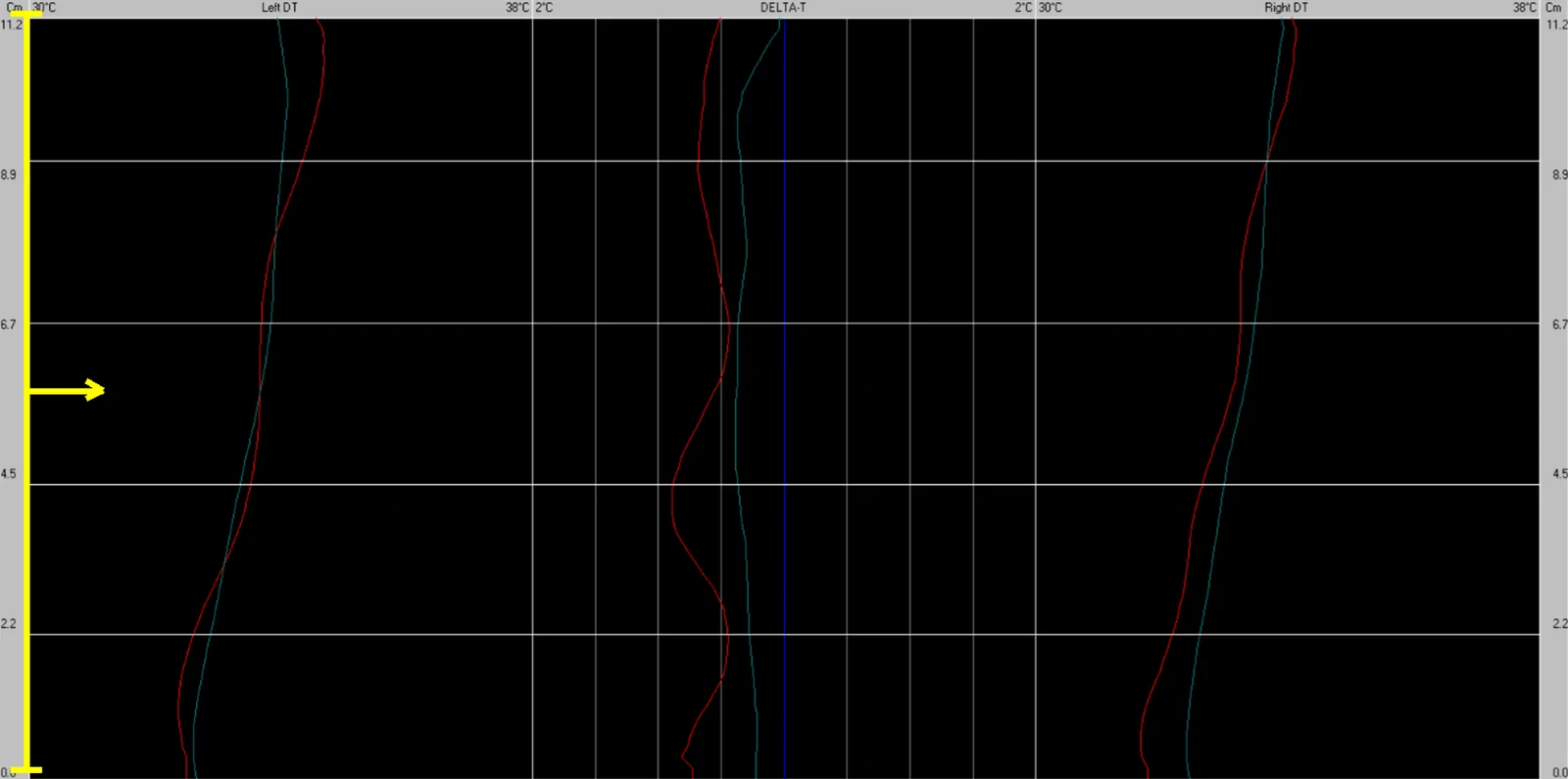
Paraspinal infrared thermography illustrating progression from baseline during the intensive week. Bracket indicates the full scan area assessed for deviation from delta, total width difference in paraspinal temperature, and baseline establishment. Dates and practitioner comment text have been redacted to protect confidential patient information.

At the four-month return visit, the patient reported that her headache was approximately 95% improved compared with baseline. She described improvement in the brain fog and concentration difficulty she had experienced earlier in her course, noting that this had begun to improve approximately two weeks after the completion of the initial intensive program, and reported that her color discrimination had also improved. No adverse reactions or side effects were reported at any point during care.

Patient perspective

By the end of the one-week intensive program, the patient reported that she had regained sensation in her occipital region, which she had lost following her prior nerve-block procedure, commenting that she was now able to feel her occiput. She also described her headache as improved, along with an improved ability to tolerate using her phone and no further sensitivity to light or sound. At her four-month return, she described her headache as approximately 95% resolved and reported that her earlier difficulty with focus and mental clarity, as well as her color-vision changes, had improved.

Final outcome

Following the completion of care in Singapore, the patient was referred to and now receives maintenance care from a full-spine chiropractor in Sri Lanka.

Adverse events

No adverse events were observed in this case. Broader prospective cohort data on the safety of upper cervical chiropractic care have been reported elsewhere [8]; this single case cannot itself establish or generalize to a safety profile.

## Discussion

This case describes a patient with a long-standing history of migraine who was diagnosed with status migrainosus during a hospital admission and who separately described her headache pattern using the term "cluster headache" of unconfirmed clinical provenance. She underwent a short-duration, intensive course of KCUC chiropractic care necessitated by international travel, showing a temporal association between this care and improvement in headache severity, photophobia/phonophobia, and several objective postural and orthopedic examination findings.

The rationale for directing manual care at the upper cervical spine in patients with primary headache disorders rests, in part, on electrophysiological evidence that trigeminal and upper cervical afferents converge on shared second-order neurons in the trigeminocervical complex, providing a plausible mechanism by which cervical afferent input could modulate headache-related nociception [3]. However, the clinical evidence for manual and chiropractic therapy in headache disorders is mixed and should be interpreted cautiously. A recent network meta-analysis found cervical spine manipulation to compare favorably with mobilization, exercise, and massage for short-term pain and disability outcomes in cervicogenic headache specifically [9], while a separate systematic review focused on chiropractic spinal manipulation across cervicogenic, tension-type, and migraine headache concluded that the certainty of evidence for its effectiveness remained very low [10]. This case is reported as a single, temporally associated clinical observation and is not presented as evidence of the efficacy of KCUC care for cluster headache, migraine, status migrainosus, or any other headache diagnosis.

It is notable that this patient's headache history includes one clinician-documented diagnosis, status migrainosus (ICHD-3 code 1.4.1, a single, debilitating migraine attack unremitting for more than 72 hours, diagnosed during her hospitalization (day -30 to day -28)), and one patient-applied label, "cluster headache" (a term describing a trigeminal autonomic cephalalgia with shorter, cyclical, strictly unilateral attacks and ipsilateral autonomic features), whose clinical source could not be confirmed from the available records [1,2]. We are not in a position to determine whether a clinician actually applied the cluster headache label, whether the patient arrived at it through her own research, or whether her presentation genuinely reflects overlapping migraine and cluster headache features, a recognized, if uncommon, clinical phenomenon sometimes discussed in the literature as "cluster migraine" [11]. Given this uncertainty, we treat "cluster headache" as an unconfirmed differential rather than a diagnosis, and we do not weight it equivalently to the documented status migrainosus diagnosis anywhere in this report. We report both possibilities as part of the patient's full clinical picture, together with her decade-long background of migraine, rather than adjudicating between them ourselves.

The neurological symptom cluster experienced by this patient, confusion, memory difficulty, color-discrimination difficulty, speech fatigue, and limb weakness, is atypical for migraine, status migrainosus, or cluster headache in isolation, and none of these symptoms were documented at the time of the hospitalization itself. It is notable that the only neuroimaging documented in this patient's history (brain MRI, approximately two years before day 0; head and neck MRI/CT, approximately one year before day 0) predates this later episode and was not obtained to investigate it; these studies therefore cannot be taken as having excluded a structural, vascular, or ischemic cause for these symptoms, and we make no claim that such causes were excluded. This includes, but is not limited to, hypertensive encephalopathy (notable given an admission blood pressure of 165/109 mmHg), meningitis, and other infective or structural causes; none of these are documented as having been explicitly excluded in the records available to us. The underlying cause of this symptom cluster cannot be determined from the available records. We do not attribute its resolution to the chiropractic care described in this report; it is included for completeness in line with CARE reporting guidance and to give an accurate account of the patient's full clinical course.

Examination findings consistent with cervicogenic contribution to headache, postural asymmetry, restricted cervical range of motion, and asymmetric weight-bearing, are not pathognomonic for any single headache diagnosis and can coexist with primary headache disorders without establishing causation. Similarly, infrared thermography was used in this case both to help guide the timing of repeated adjustments during an unusually compressed care schedule and, descriptively, as a clinical outcome measure (Figure 7), reflecting its established use at the treating clinic. This use should be interpreted cautiously: the research evidence supporting thermography as a validated, reliable outcome measure for headache disorders remains thin, and no quantitative thermographic findings are reported here, only qualitative, visually compared scan images.

The condensed, twice-daily care model used here differs from the treatment frequency described in most prior KCUC case reports and was adopted specifically to accommodate the logistical constraints of a patient traveling internationally for care, with reassessment before each adjustment used to avoid over-treatment. Whether this intensive, short-duration format offers any advantage or disadvantage relative to more conventional, lower-frequency care schedules cannot be determined from a single case and would require comparative study.

Several factors beyond KCUC care itself could plausibly account for the improvement reported in this case and cannot be excluded. These include the delayed anti-inflammatory effect of the corticosteroid occipital nerve block administered during hospitalization; continued pharmacological migraine prophylaxis and abortive therapy; the natural fluctuating course of migraine and status migrainosus, including regression to the mean; placebo or other contextual effects associated with an intensive, high-contact care program; non-specific factors associated with international travel, including environmental and climate change; and the concurrent functional neurological (vestibulo-ocular) exercises administered during the same visits, whose contribution cannot be isolated from that of the chiropractic adjustments. This case cannot distinguish among these possibilities and a treatment effect specific to KCUC.

Limitations

This case is subject to several important limitations.

As a single case report, no causal inference can be drawn between KCUC care and the patient's symptomatic improvement; temporal association does not establish efficacy. Alternative explanations for the improvement, including the occipital nerve block, continued medication, concurrent functional neurological exercises, the natural course of migraine and status migrainosus, regression to the mean, and placebo or contextual effects, are discussed above and cannot be excluded (see Discussion).

A discharge or follow-up BQ score was not obtained; outcome at follow-up is therefore supported by clinician-observed examination findings and the patient's unstructured self-report rather than a validated, re-administered outcome measure.

Thermography was used both to guide care timing and, descriptively, as a clinical outcome measure (Figure 7), consistent with its established use at the treating clinic; however, the research evidence supporting thermography as a validated, reliable outcome measure for headache disorders remains thin, and only qualitative, visually compared images, not quantitative data, are reported.

Examination methods used in this case (e.g., red desaturation, smooth pursuit, prone leg length check) do not have standardized, validated positive-finding criteria comparable to those in other medical specialties, and no formal inter-examiner reliability testing was performed; all findings were obtained by a single examiner.

The clinical relevance of postural, leg-length, and weight-distribution findings to headache outcomes specifically is uncertain; their use to guide adjustment timing in this case should not be read as validating them as headache outcome measures.

Functional neurological exercises were administered concurrently with KCUC adjustments; the independent contribution of the chiropractic care to the outcomes reported cannot be isolated from that of the exercises.

The four-month follow-up assessment relied on patient self-report of symptom improvement without in-person re-examination of all baseline findings, introducing potential recall bias.

The patient's headache diagnosis is not fully resolved in the available records. Her treating consultant neurologist documented status migrainosus at hospitalization, and this is treated as confirmed. The patient separately described her headache pattern using the term "cluster headache", but it could not be established whether this label originated from a clinician's assessment or from the patient's own research; it is reported here only as an unconfirmed differential, not as a diagnosis, and this report does not exclude the possibility that her presentation is more fully explained by migraine and status migrainosus alone.

Incomplete phenotyping of the headache pattern described as "cluster headache" (attack duration, periodicity, and associated autonomic signs were not documented) limits confidence in any diagnostic classification discussed in this report.

No neuroimaging or other investigation specific to the post-discharge neurological symptoms (confusion, memory difficulty, limb weakness) is documented in the available records; the only imaging on file (brain MRI approximately two years before day 0, head/neck MRI/CT approximately one year before day 0) predates these symptoms and cannot be relied upon to exclude a structural or vascular cause for them. The underlying cause of these symptoms could not be independently verified by the chiropractic authors. The patient was verbally advised to follow up with her neurologist regarding these findings; as she already had an established treating neurologist, no formal referral letter was issued by the chiropractic authors.

Red-flag differentials for the post-discharge neurological symptoms, including hypertensive encephalopathy, meningitis, and other infective or structural causes, are not documented as having been explicitly excluded in the records available to the chiropractic authors; any further investigation would have been at the discretion of the treating hospital medical team.

The patient was lost to structured follow-up after her return to Sri Lanka, limiting longer-term outcome data.

## Conclusions

This case describes a temporal association between a short-duration, intensive KCUC chiropractic care model and improvement in headache burden and cervical examination findings in a patient with a 10-year history of migraine who was diagnosed with status migrainosus during a hospital admission and who separately described her headache pattern using the term "cluster headache", a label of unconfirmed clinical provenance reported here as a differential rather than an established diagnosis. Her course was also complicated by a hospitalization and, in the week following discharge, a cluster of transient neurological symptoms for which no contemporaneous neuroimaging is documented; these symptoms and their underlying cause remain incompletely characterized in the available records and are not attributed to the chiropractic care described here. The case adds to a limited literature on condensed-format upper cervical care for traveling patients but should be interpreted only as a single clinical observation. Given the low certainty of existing evidence for chiropractic manual therapy across headache subtypes, and the diagnostic uncertainty in this patient's headache classification, further prospective, controlled research using validated, consistently re-administered outcome measures is needed before conclusions can be drawn about the effectiveness of this approach.
